# Delayed rhegmatogenous retinal detachment following high-voltage electrical injury: a case report

**DOI:** 10.3389/fmed.2026.1831902

**Published:** 2026-04-15

**Authors:** Chaoxiong Cui, Danyang Yu, Ying Sun, Feng Teng, Shuzhen Wang

**Affiliations:** Department of Ophthalmology, Qingdao Central Hospital, University of Health and Rehabilitation Sciences, Qingdao, Shandong, China

**Keywords:** electric cataract, electrical injury, pars plana vitrectomy, rhegmatogenous retinal detachment, vitreoretinal complications

## Abstract

**Background:**

Electric cataract is a recognized complication of high-voltage electrical injury, resulting from direct electrical and thermal damage to ocular tissues. Although cataract formation is the most commonly reported manifestation, electrical injury should be regarded as a pan-ocular insult capable of affecting both anterior and posterior segment structures. Delayed posterior segment complications, particularly rhegmatogenous retinal detachment (RRD), remain poorly characterized and may be overlooked during early management.

**Case presentation:**

We report the case of a patient who developed unilateral electric cataract following a high-voltage electrical injury. During primary surgery, posterior capsule rupture with complete posterior dislocation of the lens nucleus was observed, and pars plana vitrectomy (PPV) with lensectomy was performed. Despite initially favorable postoperative recovery, the patient presented 86 days later with macula-involving RRD characterized by multiple retinal breaks and star-shaped retinal folds. A secondary PPV combined with epiretinal membrane peeling, endolaser photocoagulation, and silicone oil tamponade was undertaken, resulting in successful retinal reattachment. After silicone oil removal, best-corrected visual acuity improved and stabilized at 0.5, with the retina remaining flat during follow-up.

**Conclusion:**

This case highlights that electrical ocular injury represents a dynamic and progressive process rather than a static event confined to the time of trauma. Successful management of anterior segment pathology does not preclude the development of delayed, sight-threatening posterior segment complications. Long-term, vigilant vitreoretinal surveillance is essential in patients with electrical eye injuries to enable early detection and timely intervention, thereby preventing irreversible vision loss.

## 1 Introduction

Electric cataract is a common sequela of high-voltage electrical injury (1, 2). The lens is highly sensitive to electrical and thermal insults due to its high protein density and poor regenerative capacity, resulting in progressive opacification (3–5). While cataract formation is the primary clinical finding, high-voltage trauma often causes pan-ocular damage, affecting both the anterior and posterior segments (6–8). Thermal energy and metabolic disruption of lens epithelial cells contribute to this process, frequently leading to secondary damage of the lens capsule and zonular integrity (9). Despite successful primary cataract surgery, patients remain at risk for delayed posterior segment complications, including complex vitreoretinal pathologies that require long-term monitoring (10).

In this report, we describe a 58-year-old male who presented 6 months after a high-voltage electrical injury. At the time of admission, the patient reported a progressive decline in visual acuity of the left eye over the preceding 4 months. Physical examination revealed extensive hypertrophic scarring on the left upper eyelid and cheek from previous skin grafts. Ophthalmic evaluation showed a best-corrected visual acuity (BCVA) of hand motion (HM) in the left eye. Slit-lamp examination identified diffuse grayish-white cortical opacification and anterior capsular wrinkling. Ancillary imaging, specifically B-scan ultrasonography, revealed an oval echogenic mass within the vitreous cavity, raising strong suspicion of posterior lens dislocation—a finding subsequently confirmed intraoperatively. A critical feature of this case was the delayed onset of rhegmatogenous retinal detachment (RRD) with Grade C proliferative vitreoretinopathy (PVR) occurring 86 days post-lensectomy. This clinical trajectory underscores the dynamic evolution of electrical ocular trauma and highlights the mandatory requirement for long-term posterior segment surveillance.

## 2 Case presentation

A 58-year-old male, working as a daily manual laborer, was admitted to the Department of Ophthalmology, Qingdao Central Hospital, on May 27, 2024, with a six-month history of progressive visual decline in the left eye, which had worsened over the preceding 4 months. Six months prior to presentation, he sustained a high-voltage electrical injury at work after accidental contact with an inadequately insulated electrical device using his left hand. The exact voltage was unknown. According to the witnesses present at the scene of the accident, there was no history of direct cranial or ocular blunt trauma, such as a fall or impact, during the electrical injury. He was immediately transferred to the emergency department and subsequently managed in the Burns Department, where he underwent left forearm amputation and multiple reconstructive skin graft procedures involving the head and face. The skin graft area partially extended to the left upper eyelid and cheek. There was no remarkable personal, marital, or family medical history.

On ophthalmic examination, best-corrected visual acuity (BCVA) was 0.8 in the right eye (−0.50 DS/−0.25 DC × 65) and hand motion before the eye (HM/BE) in the left eye. Intraocular pressure (IOP) was 14 mmHg in both eyes. The right eye was unremarkable. Examination of the left eye revealed hypertrophic scarring of the upper eyelid with skin graft involvement, resulting in cicatricial ptosis partially covering the pupil. Mild cicatricial ectropion of the lower eyelid and conjunctival hyperemia were observed secondary to cheek scarring. On eyelid closure, approximately 1 mm of scleral show was noted; Bell’s phenomenon was intact.

**Figure 1:**
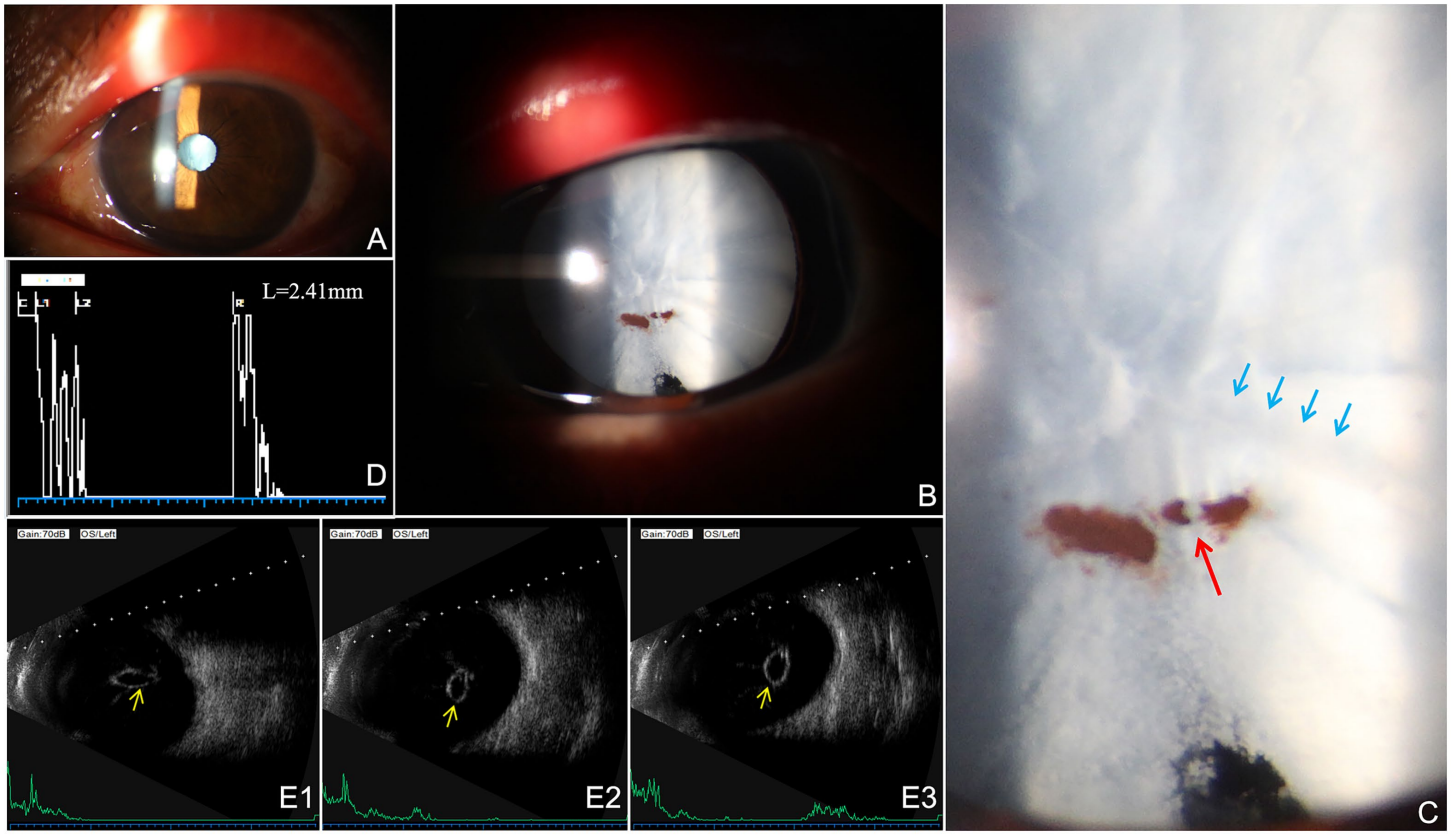
Preoperative multimodal imaging of the left eye. **(A,B)** Anterior segment photographs: Slit-lamp biomicroscopy reveals a mature electric cataract with dense cortical opacification. **(C)** Magnified view of the anterior capsule. A distinct gap in the continuity of iris pigment deposits is noted (red arrow). Parallel linear shadowing is projected from the wrinkled capsule onto the underlying lens cortex (blue arrows), providing structural evidence of capsular wrinkling and zonular instability. **(D)** A-scan ultrasonography waveforms: Following dislocation of the lens nucleus into the vitreous cavity, the lens thickness of the left eye was markedly reduced. **(E1–E3)** The B-scan ultrasonography: Serial B-scan ultrasonography identifies an ovoid echogenic mass (yellow arrows) within the vitreous cavity, with no evidence of retinal detachment or peripheral breaks.

Slit-lamp examination of the left eye demonstrated a clear cornea and a moderately deep anterior chamber. Mild iridodonesis was present. The pupil was round (2.5 mm) with preserved light reflex. The anterior capsule was notably wrinkled, showing focal brown pigment spots inferiorly. Interestingly, these pigment deposits exhibited a clear gap in their continuity (Figure 1C, red arrow), and a distinct linear shadow was projected from the wrinkled capsule onto the underlying cortex (Figure 1C, blue arrows). The lens also showed diffuse, layered grayish-white cortical opacification occupying nearly the entire pupillary zone (Figures 1A,B). The lens nucleus and posterior capsule were not visible, and mild lens mobility was detected. Fundus examination was not possible due to media opacity.

In the left eye, serial B-scan ultrasonography identified an oval echogenic mass in the vitreous cavity (Figures 1E1–E3, yellow arrows). Multi-quadrant scanning showed the retina to be entirely flat, with no visible peripheral breaks or vitreoretinal traction, providing a continuous longitudinal and transverse assessment of the posterior segment. A-scan biometry demonstrated axial lengths of 23.08 mm in the right eye and 23.09 mm in the left eye. The anterior chamber depth (ACD) was 2.97 mm in the right eye and 3.76 mm in the left eye. Lens thickness measured 4.79 mm in the right eye but only 2.41 mm in the left eye (Figure 1D). The markedly reduced lens thickness in the left eye was considered artifactual and likely attributable to posterior dislocation of the lens nucleus into the vitreous cavity, resulting in underestimation of the remaining lenticular structure on biometry. Based on the anterior segment findings, biometric measurements, and ultrasonographic features, posterior dislocation of the lens nucleus into the vitreous cavity was suspected. The preliminary diagnoses were electric cataract (OS), lens subluxation (OS).

After routine preoperative topical antibiotic prophylaxis, pars plana vitrectomy (PPV) combined with lensectomy and intraocular lens (IOL) implantation was performed in the left eye on May 28, 2024. Intraoperatively, a vertical rupture of the posterior capsule was observed, with complete posterior dislocation of the lens nucleus into the vitreous cavity, while the anterior capsule remained intact. A standard three-port PPV was performed using the Constellation system (Alcon Laboratories, United States). The vitreous body was removed, and the dislocated lens nucleus was extracted using a vitreous cutter under perfluorocarbon liquid protection. Residual cortical material beneath the anterior capsule was carefully removed. To minimize postoperative capsular contraction and enhance IOL stability, a limited central anterior capsulotomy was performed. Peripheral retinal examination revealed no abnormalities. A four-haptic foldable posterior chamber IOL (Akreos Adapt AO + 20.5 D, Bausch & Lomb, United States) was implanted in the ciliary sulcus and positioned horizontally after pharmacologic pupil constriction. Following IOL implantation, the peripheral retina was examined 360 degrees under direct visualization with the vitrectomy system. With the aid of concurrent scleral depression, the entire retinal periphery was verified to be flat, with no iatrogenic breaks, lattice degeneration, or visible vitreoretinal traction identified at the conclusion of the procedure. The intraoperative findings confirmed that the echogenic lesion observed preoperatively on ultrasonography corresponded to the dislocated lens nucleus. Postoperatively, a standardized regimen of topical antibiotics, corticosteroids, and non-steroidal anti-inflammatory agents was administered (Table 1).

**Table 1:** Timeline of clinical events, diagnosis, and therapeutic interventions.

Time point	Key clinical events and findings	Interventions and management
6 months prior to admission	Sustained high-voltage electrical injury at work; no history of direct ocular or cranial blunt trauma.	Initial systemic stabilization; left forearm amputation and facial reconstructive skin grafts.
Initial presentation (May 27, 2024)	Progressive visual decline (OS: HM/BE); cataract with iris pigment spots and anterior capsule wrinkling. Diagnosis of electric cataract and posterior lens nucleus dislocation.	Comprehensive ophthalmic evaluation: B-scan (oval echogenic mass), Biometry (deepened ACD, artifactual lens thinning).
Day 1 after admission (May 28, 2024)	Intraoperative Observation: Vertical rupture of the posterior capsule with complete nuclear dislocation. Anterior capsule remained intact.	Primary Surgery: PPV with lensectomy and sulcus-fixated IOL implantation.
Postoperative day 1	Improved BCVA (0.25); stable IOL centration; retina remained	Post-op meds: Levofloxacin, Pranoprofen, and Tobramycin–dexamethasone drops (QID, OS)
Postoperative week 1	Improved BCVA (0.5); stable IOL centration; retina remained completely flat on fundus examination.	Post-op meds: Levofloxacin, Pranoprofen, and Tobramycin–dexamethasone drops (TID, OS)
86 days post-primary surgery	Sudden visual obstruction (OS: HM/BE); total RRD involving the macula.	Identified star-shaped retinal folds, PVR Grade C, and round retinal breaks. Confirmed by B-scan and OCT.
September 3, 2024	Progression of PVR and secondary retinal detachment.	Secondary Surgery: PPV with epiretinal membrane peeling, endolaser photocoagulation, and silicone oil tamponade. Post-op meds: Levofloxacin, Pranoprofen, and Tobramycin–dexamethasone drops (QID, OS)
Follow-up (8 months)	Stable retinal reattachment under silicone oil; BCVA maintained at 0.15.	Periodic monitoring of IOP and retinal stability.
May 27, 2025 (one year after admission)	Retina remained attached; no signs of further PVR progression.	Silicone Oil Removal: Performed one year after the initial presentation. Post-op meds: Levofloxacin, Pranoprofen, and Tobramycin–dexamethasone drops (QID, OS)
Final follow-up (3 months post-oil removal)	Sustained retinal attachment; final BCVA improved to 0.5.	Case concluded with successful anatomical and functional recovery.

**Figure 2:**
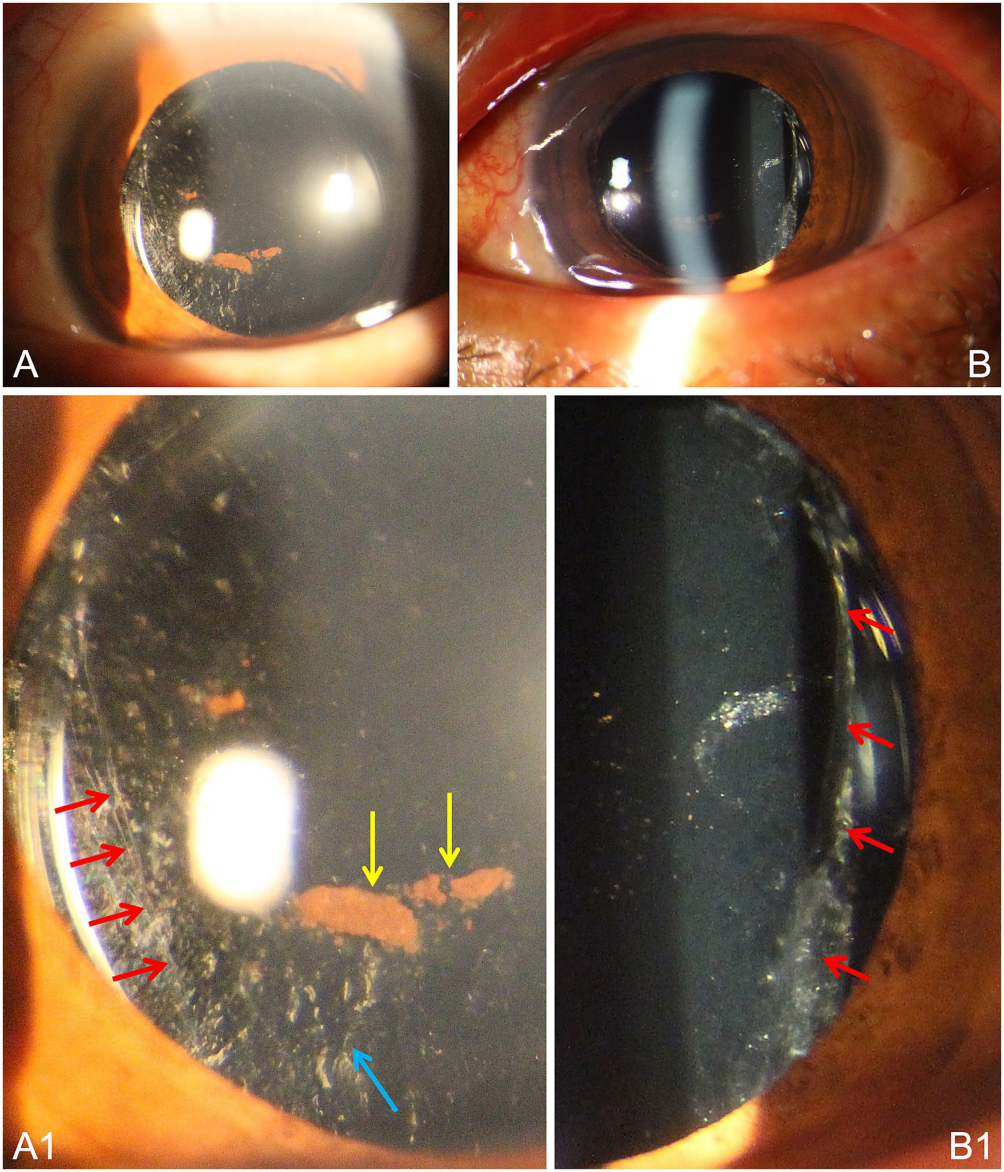
Anterior segment photographs after PPV combined with lensectomy and IOL implantation of the left eye. **(A,B)** Slit-lamp overview postoperatively showing a stable IOL well positioned in the ciliary sulcus. **(A1)** Inferonasal view. Residual iris pigment (yellow arrows) and wavy capsular wrinkling (blue arrow) are seen on the preserved anterior capsule. The edge of the perforated posterior capsule is also partially visible (red arrows). **(B1)** Temporal view. High-magnification visualization of the perforated posterior capsule edge (red arrows) adjacent to the IOL optic, providing morphological proof of the primary electric injury.

On postoperative day one, BCVA in the left eye was 0.25, and IOP was 12 mmHg. The incision was well sealed. Mild corneal edema with Descemet membrane folds was noted. The anterior chamber was of normal depth with mild cellular reaction. The IOL was stable and centered, supported by the residual peripheral capsule (Figure 2). Slit-lamp examination identified the preoperative iris pigment (yellow arrows) and wavy wrinkling (blue arrow) on the residual anterior capsule, alongside the irregular edge of the perforated posterior capsule (red arrows). Fundus examination was unremarkable. The patient was discharged on postoperative day one. At the one-week follow-up, BCVA improved to 0.5 (+0.25 DS/−3.75 DC × 15), IOP was 9.5 mmHg, and the IOL remained stable. Fundus examination demonstrated a flat retina with no hemorrhage or exudation. The 1-month follow-up was missed as the patient, a manual laborer, underestimated the importance of ophthalmic surveillance after experiencing a dramatic visual recovery (from HM/BE to 0.5). He remained on medical leave throughout this interval with no history of heavy lifting or physical exertion.

**Figure 3:**
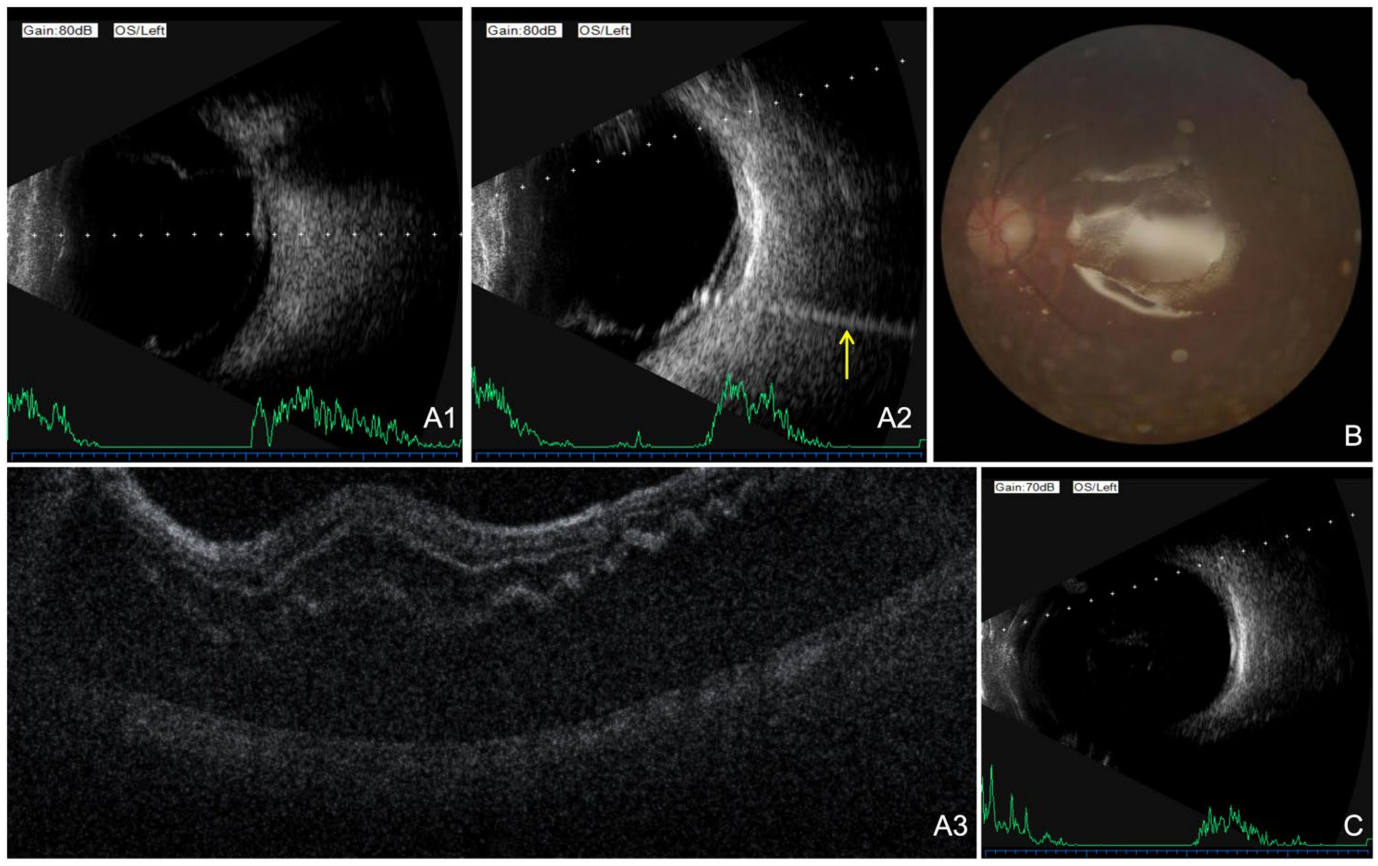
Multimodal imaging of rhegmatogenous retinal detachment (RRD) and outcomes after silicone oil removal of the left eye. **(A1,A2)** B-scan ultrasonography: Ultrasound revealed a V-shaped hyperechoic band in the vitreous cavity, attached to the optic disc, consistent with retinal detachment (yellow arrow: a linear acoustic artifact). **(A3)** Optical coherence tomography: The retinal neuroepithelium and the retinal pigment epithelium were separated in the scanned region. The internal architecture of the neuroepithelium was poorly defined, with visible undulating folds. **(B)** Color fundus photography: Silicone oil occupied the vitreous cavity, forming a well-defined reflective interface, with the retina remaining flat. **(C)** B-scan ultrasonography: Follow-up B-scan after silicone oil removal. Several highly echogenic silicone oil droplets were detected in the vitreous cavity, and the retina remained well attached.

Eighty-six days after the primary intraocular surgery, approximately 6 months after the electrical injury, the patient reported sudden visual obstruction in the left eye. Visual acuity had declined to HM/BE. Indirect ophthalmoscopy revealed inferior and nasal rhegmatogenous retinal detachment involving the macula, with grayish retinal elevation. Star-shaped retinal folds were observed at the posterior pole, adjacent to a round retinal break. An additional retinal break approximately one-third disc diameter in size was identified near the superotemporal ora serrata. B-scan ultrasonography (Figures 3A1,A2) and OCT (Figure 3A3) confirmed the total RRD and macular involvement.

On September 3, 2024, secondary PPV with epiretinal membrane peeling, endolaser photocoagulation, and silicone oil tamponade was performed. Intraoperatively, the vitreous was completely removed, and the epiretinal membrane corresponding to the star-shaped retinal folds was peeled. Perfluorocarbon liquid was used to flatten the detached retina, followed by meticulous vitreous base shaving under scleral indentation. Endolaser photocoagulation was applied around all retinal breaks, and 4.0 mL of silicone oil was injected.

Postoperatively, visual acuity improved to counting fingers at 15 cm and to 0.15 with pinhole correction. IOP was 16 mmHg. The IOL remained stable in the ciliary sulcus, and the silicone oil was *in situ*. Fundus examination confirmed complete retinal reattachment with well-demarcated laser scars (Figure 3B). At the eight-month follow-up, the retina remained attached under silicone oil tamponade, with a BCVA of 0.15 and IOP of 13 mmHg. Silicone oil removal was performed on May 27, 2025. One week later, BCVA improved to 0.3, IOP was 18 mmHg, and the retina remained flat (Figure 3C). At one- and three-month follow-up visits after silicone oil removal, BCVA further improved to 0.5, with sustained retinal attachment.

## 3 Discussion and conclusions

The eye provides a low-resistance pathway for electrical current due to the high water and electrolyte content of intraocular tissues (11). In this case, the intraoperative observation of a ruptured posterior capsule and complete posterior dislocation of the lens nucleus aligns with substantial structural weakening of the capsular bag following high-voltage exposure. Such damage predisposes the lens to acute or delayed instability rather than isolated opacification (12, 13). The unilateral involvement observed here is consistent with the probable current pathway, which entered through the left upper limb and exited via the ipsilateral facial region.

The most notable aspect of this case is the delayed onset of RRD, occurring approximately 86 days after the initial lensectomy. While electric cataracts are well-documented, the subsequent development of RRD following high-voltage injury is relatively rare and often characterized by a deceptive latency period (3). We propose that the pathophysiology of this delayed RRD is multifactorial. The high-voltage current likely induced immediate but subclinical thermal insult to the peripheral retina and vitreous base. This “electroporation” effect, combined with localized thermal damage, potentially triggered premature vitreous liquefaction and abnormal vitreo-retinal traction that evolved progressively over several weeks (14). Furthermore, the electrical trauma might have caused a chronic breakdown of the blood-aqueous barrier or subtle ciliary body dysfunction, leading to persistent low-grade intraocular inflammation that further compromised retinal structural integrity (15). It is crucial to differentiate this delayed RRD from potential iatrogenic or post-vitrectomy changes. In this case, the peripheral retina was examined 360 degrees with scleral depression at the conclusion of the primary surgery, which confirmed a flat retina with no visible iatrogenic breaks or pre-existing lesions. The 86-day latency, combined with the rapid progression of Grade C proliferative vitreoretinopathy (PVR), aligns with the documented timeline of progressive vitreoretinal remodeling following high-voltage insult rather than typical acute surgical complications (10). These findings suggest that the retinal detachment was a dynamic, delayed consequence of the initial electrical trauma rather than a procedural event.

In this patient, the discovery of multiple retinal breaks and star-shaped folds (Proliferative Vitreoretinopathy Grade C) at the 86-day follow-up indicates that the retinal damage was a dynamic, progressive process rather than a static event. The lens dislocation itself served as early evidence of severe zonular and capsular frailty, which likely contributed to unstable intraocular dynamics post-operatively (16). PPV with epiretinal membrane peeling, endolaser photocoagulation, and silicone oil tamponade remains the standard approach for such complex traumatic RRD (18). In our case, timely intervention resulted in stable retinal reattachment and a final BCVA of 0.5. Although intraoperative recording was limited by equipment, the consistency between surgical findings, postoperative imaging, and functional recovery supports the clinical interpretation.

In conclusion, electrical ocular injury must be managed as a progressive condition. Successful anterior segment surgery should not be considered the end of treatment; instead, it must be followed by long-term posterior segment surveillance to facilitate early detection and management of delayed, sight-threatening complications (17).

## Data Availability

The original contributions presented in the study are included in the article/supplementary material, further inquiries can be directed to the corresponding author.
